# Evaluation of a novel MRI-safe needle guidance toolkit for streamlined arthrography procedures

**DOI:** 10.1038/s41598-023-45063-w

**Published:** 2023-10-17

**Authors:** Reza Monfaredi, Pavel Yarmolenko, Eung-Joo Lee, Kevin Cleary, Karun Sharma

**Affiliations:** 1 Sheik Zayed Institute for Pediatric Surgical Innovation, Children’s National Hospital, Washington, DC USA; 2 Department of Electrical and Computer Engineering, University of Arizona, Tucson, AZ USA

**Keywords:** Engineering, Biomedical engineering, Musculoskeletal system

## Abstract

Currently, Magnetic Resonance arthrography procedures require two rooms and two imaging modalities: fluoroscopically guided needle insertion in a fluoroscopy suite, followed by diagnostic MRI in a separate MRI suite. The use of fluoroscopy for needle placement exposes patients to ionizing radiation, which is an important concern, especially in pediatrics. The need for two different rooms and coordinating times for these rooms complicates hospital resource scheduling and logistics. In addition, the added delays could expose younger children to additional risks associated with the use of general anesthesia. To address these issues, we propose a new technique to streamline the arthrography procedure. Our proposed technology aims to eliminate exposure to ionizing radiation and to streamline arthrography procedures that are conducted solely under MRI. This toolkit consists of a 3D slicer-based user interface, a spatially unique silicone grid template, and a hand-held needle guidance device. Together, these tools are intended to simplify and shorten the procedure while maintaining accuracy and precision comparable to the current gold standard procedure. In our cadaver study, we evaluated the feasibility and accuracy of our novel MRI-safe Needle Guidance Toolkit for MRI arthrography procedures, achieving an average targeting accuracy of 3.2 ± 1.0 mm. The results presented in this study showed the feasibility and promise of our novel MRI-safe needle guidance toolkit for arthrography procedures.

## Introduction

Arthrography is a commonly performed procedure that is used to evaluate pathological conditions and injuries such as ligamentous and cartilage tears that occur within the joints of the body. Such problems are common for people of all ages, often due to aging in older adults and due to sports injuries in younger adults and children. For example, there were at least 17,000 arthrograms of the knee, 10,000 arthrograms of the hip, and 20,000 arthrograms of the shoulder performed in 2022 only in the US among the elderly based on the Medicare database^1^. Among American children ages 5–14, there is, on average, at least one sports-related injury, often involving internal derangements of their shoulders, hips, wrists, and other joints^2^. MRI arthrography is the modality of choice for the evaluation of these injuries.

Currently, the MRI arthrography procedure requires two separate rooms and coordination and transfer between these rooms. The first room accommodates a fluoroscopically guided intra-articular contrast injection to distend and fill the joint space, followed by an MRI scanner room which is used to image the intra-articular anatomy and pathology within the distended joint. In this established approach, fluoroscopy is required for needle guidance to enable injection of x-ray and MRI contrast agents into the joint space, while MRI is subsequently used for diagnosis. The requisite use of fluoroscopy for needle placement exposes patients to ionizing radiation and requires longer general anesthesia in younger children due to the transportation of the patient from the fluoroscopy room to the MRI room. The current gold standard method is also sub-optimal due to delays associated with scheduling and transferring between two procedure rooms. Besides the use of anesthesia in young patients, the two-room workflow and methods for MR arthrography are consistent across different age groups.

Manual MRI-guided needle-based procedures are challenging. Most diagnostic MRI scanners are closed-bore scanners with a bore diameter of 60 cm. The physician’s access to the patient in such scanners is limited. The patient needs to be moved in and out of the scanner for imaging and needle placement, respectively. Hand-to-eye registration based on 2D MR images is another drawback of manual needle-based MRI-guided intervention. Guiding devices or robots that can safely function in the MRI environment and address these drawbacks could improve the adaption of MRI-guided procedures.

## Materials and methods

This work describes and evaluates the performance of a novel and intuitive MRI guidance toolkit for arthrography and similar needle-based procedures. This toolkit consists of a spatially unique flexible silicone grid, a hand-held device, and a 3D slicer-based user interface for the guidance of needle insertion. 3D Slicer is an open-source software platform for medical image informatics, image processing, and three-dimensional visualization^3^. For manual path planning in needle-based procedures, the radiologist will select two points, i.e. entry point and target point, that ensure a clear path from skin to the target avoiding bony structures and critical anatomies such as nerves and large blood vessels. Our MRI-visible silicone grid is flexible, so it conforms to the patient’s body shape and provides an MRI-visible reference for each point on the skin. This MRI visible reference provides guidance for planning the desired path and locating the needle tip on the skin entry point as selected by the radiologist on the MR images. This grid allows easy cross-identification of points in MRI images and visible grid/skin locations. Each component of the system will be described next.

### Flexible silicone template

**Figure 1:**
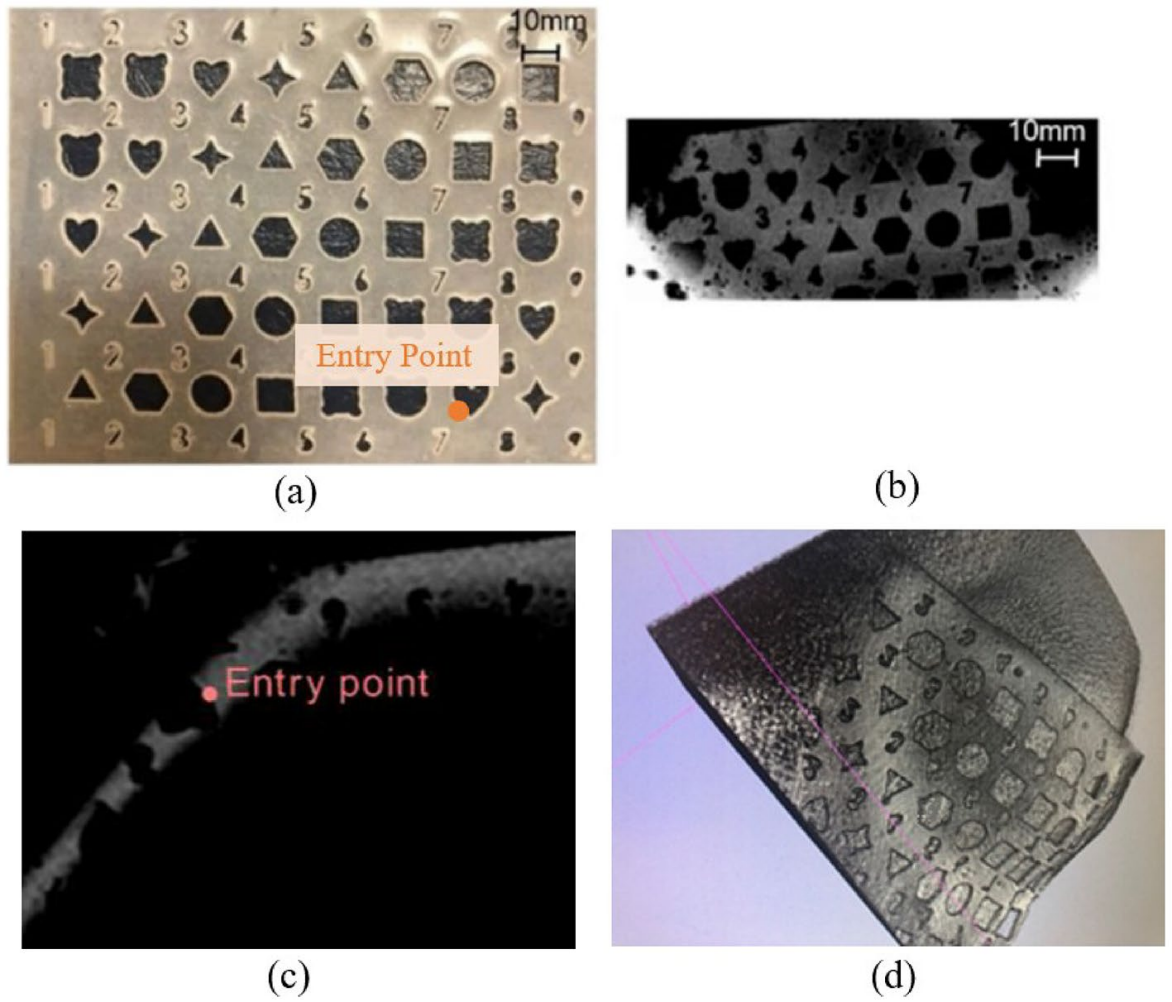
(**a**) Silicone grid template with a spatially unique, non-repeating pattern, (**b**) T1-weighted MR images of the silicone grid template, (**c**) MR image of an out-of-plane grid and a hypothetical entry point, (**d**) 3D rendered image of the silicone grid made using segmentation of the MRI dataset.

The MR-imageable, spatially unique grid template (Fig. 1a) was made using silicone (ReoFlex 40 DRY, Smooth-on Inc., PA, USA), that was polymerized in a 3D-printed mold with a spatially unique, non-repeating pattern. The mold was printed from Verowhite material using Stratasys Objet 500 3D printer. MRI-visibility of the resulting grid was tested in a 1.5 T Philips Achieva MRI system. The grid provided adequate signal intensity so that its minute features could be used to identify the planned entry point on conventional T1-weighted MR images (Fig. 1b). Figure 1c shows the image of the silicone grid when it is not in-plane with the MRI scanner’s coordinate system. A 3D-rendered image of the grid was generated using 3D Slicer’s rendering module as shown in Fig. 1d. This 3D rendered image was used to locate the entry point on the surface of the grid, providing unique local features for identification of this point both in imaging and on the grid/skin surface (Fig. 1d).

### Hand-held needle guide

**Figure 2:**
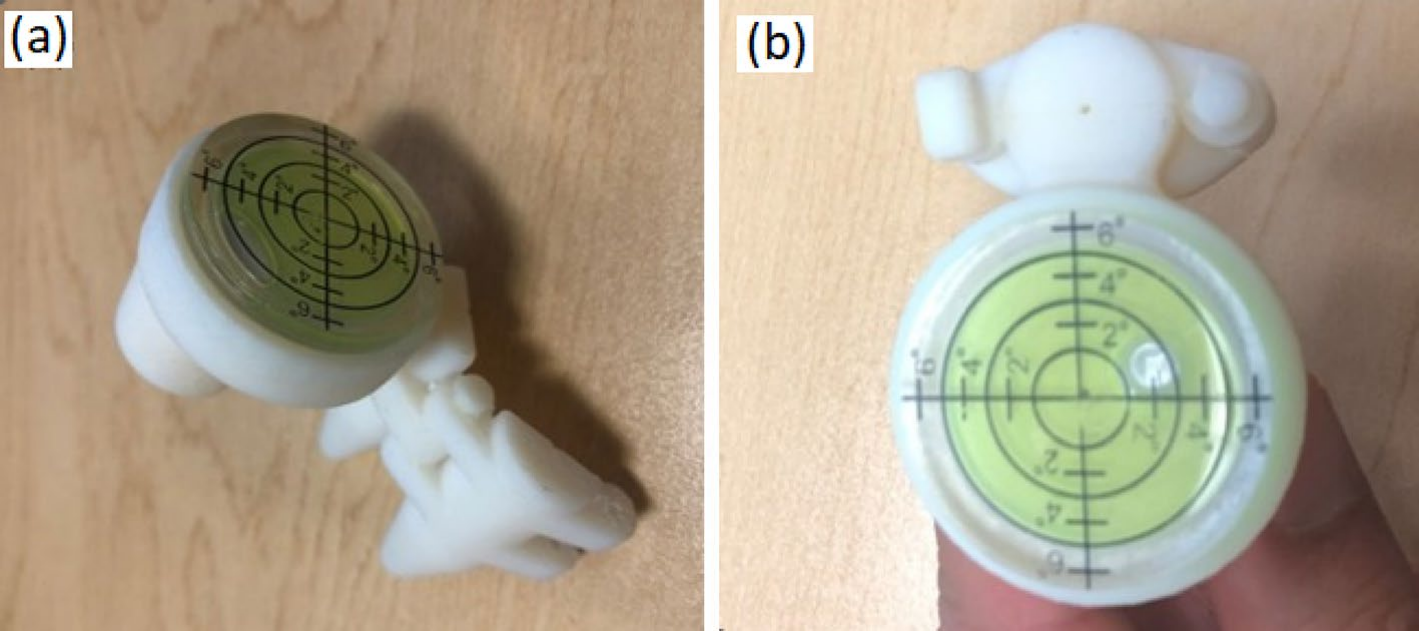
The hand-held device is shown from two different views (**a**) and (**b**), it consists of a spherical bubble level, handle, and a needle guide; the device could be held and manipulated by one or two fingers (**c**) and (**d**).

The handheld needle guide (Fig. 2) was 3D printed using VeroWhite plastic with Stratasys Objet 500 3D printer. This device consists of a spherical bubble that can measure the pitch and yaw angles (range − 6° to 6° in each direction). The needle guide was designed to have two pieces so that after needle placement the needle guide could be removed, leaving the needle in place. The silicone grid and hand-held device, in combination, allow the radiologist to position and angulate the needle properly to insert the needle and to reach the target.

### Path planning

The path planning is conducted manually by our interventional radiologist with more than 15 years of experience, based on MR images. The first step involves the selections of an entry point on the silicone template (which is conformed to the skin) and a target point within the joint space using MRI images. Once these two points are selected. Needle angulations are calculated using the spherical coordinate system as follows.

**Figure 3:**
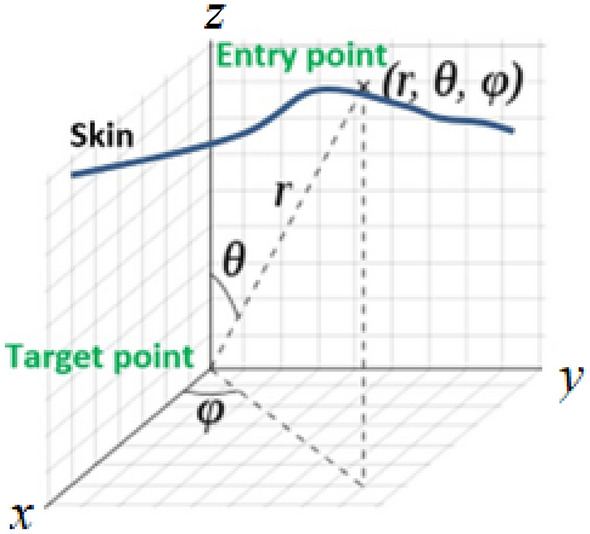
Entry and target points are shown in spherical coordinate frame.

Figure 3 shows the spherical coordinate system with the entry and target points. Needle angulation, i.e. *θ* and *φ*, could be seen as two of the three Euler angles. As shown in this figure, we assume that the origin of the spherical coordinate frame is the target point. The entry point is located in (r*, θ, φ*) with respect to the spherical coordinate frame. We assume that the needle is initially aligned along the *z*-axis so that $\overrightarrow{\mathrm{r}}=[0, 0,$ r] represents the direction of the needle in the home position. By rotating this vector about y axis by *θ* and then along the z axis by φ, $\overrightarrow{\mathrm{r}}$, will be aligned with the desired trajectory and could be represented as follows:

1 $$\overrightarrow{r}={R}_{z}(\varphi ){R}_{y}(\theta )\left(\begin{array}{c}0\\ 0\\ r\end{array}\right)$$

where the *R*_*z*_(*φ*) and *R*_*y*_(*θ*) are the rotation matrix about *z* axis and *y* axis. These rotation matrices are defined by:

2 $${R}_{z}\left(\varphi \right)=\left(\begin{array}{ccc}\begin{array}{c}cos\varphi \\ sin\varphi \\ 0\end{array}& \begin{array}{c}-sin\varphi \\ cos\varphi \\ 0\end{array}& \begin{array}{c}0\\ 0\\ 1\end{array}\end{array}\right), {R}_{y}\left(\theta \right)=\left(\begin{array}{ccc}\begin{array}{c}cos\theta \\ sin\theta \\ 0\end{array}& \begin{array}{c}-sin\theta \\ cos\theta \\ 0\end{array}& \begin{array}{c}0\\ 0\\ 1\end{array}\end{array}\right)$$

Therefore,

3 $${\overrightarrow{r}}_{x}=rcos\varphi sin\theta , {\overrightarrow{r}}_{y}=rsin\varphi sin\theta , \mathrm{and} {\overrightarrow{r}}_{z}=rcos\theta $$

Since the $\overrightarrow{\mathrm{r}}$ is known, θ and φ could be simply calculated by using Eq. (3).

### The workflow

**Figure 4:**
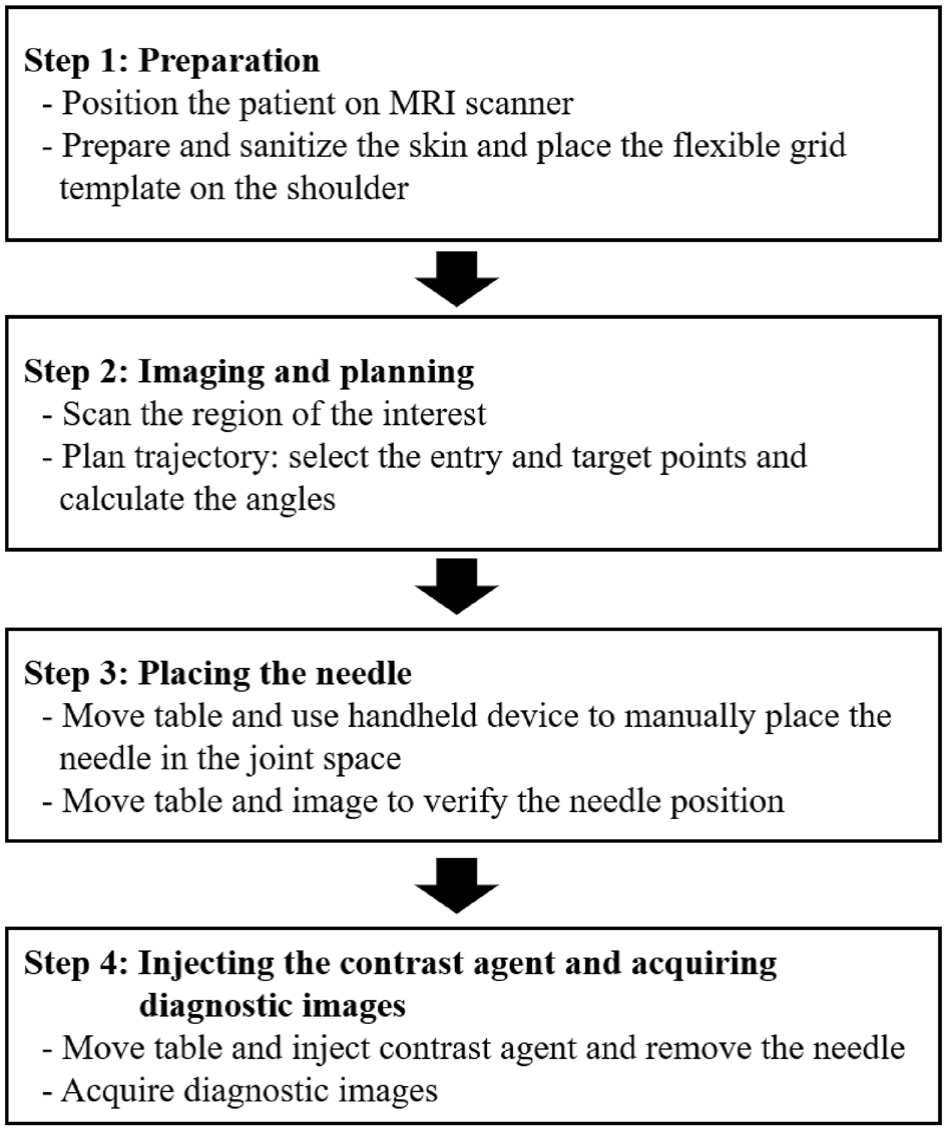
Proposed streamlined workflow.

Figure 4 shows the workflow of the arthrography procedure that is conducted for different joint examinations. The workflow is consistent across all age ranges and for all types of joints such as shoulder, hip, and ankle. This workflow consists of four main steps: (1) patient preparation, (2) MR imaging and needle path planning, (3) reproduction of the planned needle path using silicone grid and hand-held device and advancing the needle from skin entry site to the intra-articular target and confirmatory imaging, and (4) MRI contrast agent injection and imaging.

### Cadaver study

An interventional radiologist (KS) performed needle placement on a human (female, 84 years old) cadaveric specimen (Siencecare, Folcroft, Pennsylvania). The specimen was provided by Science Care Inc. Science Care Inc. is ATTB accredited and operates based on United States laws and regulations for ethical approvals.

The study was conducted in our Interventional 1.5 T Philips Achieva MRI scanner at Children’s National Hospital after receiving permission from our infection control experts. T1-weighted MR imaging (T1, resolution 1 mm, field of view (FV) 200 × 200, time of echo (TE) 1.79, time of relaxation (TR) 4.43) was used for path planning since our silicone grid is visible in T1-weighted images. For path planning, T2-weighted imaging (T2, SL4, Resolution 1 mm, FOV 200 × 200, TE 57, TR 3300) was used since this imaging sequence provides better anatomical imaging quality for visualization of the joint space for path planning. In clinical cases, the contrast agent is injected within the joint space to examine the condition of the joint and diagnose an injury or other pathology within the joint. The goal of this study was to investigate the feasibility and targeting accuracy only. Since we were not expecting any injury of the shoulder joint, we did not perform step 4 of the workflow during this cadaver study.

### Ethics approval and consent to participate

The specimen study was according to Children’s National Ethics approval requirement and provided by Science Care Inc. which is ATTB accredited and operates based on United States laws and regulations.

## Results

**Figure 5:**
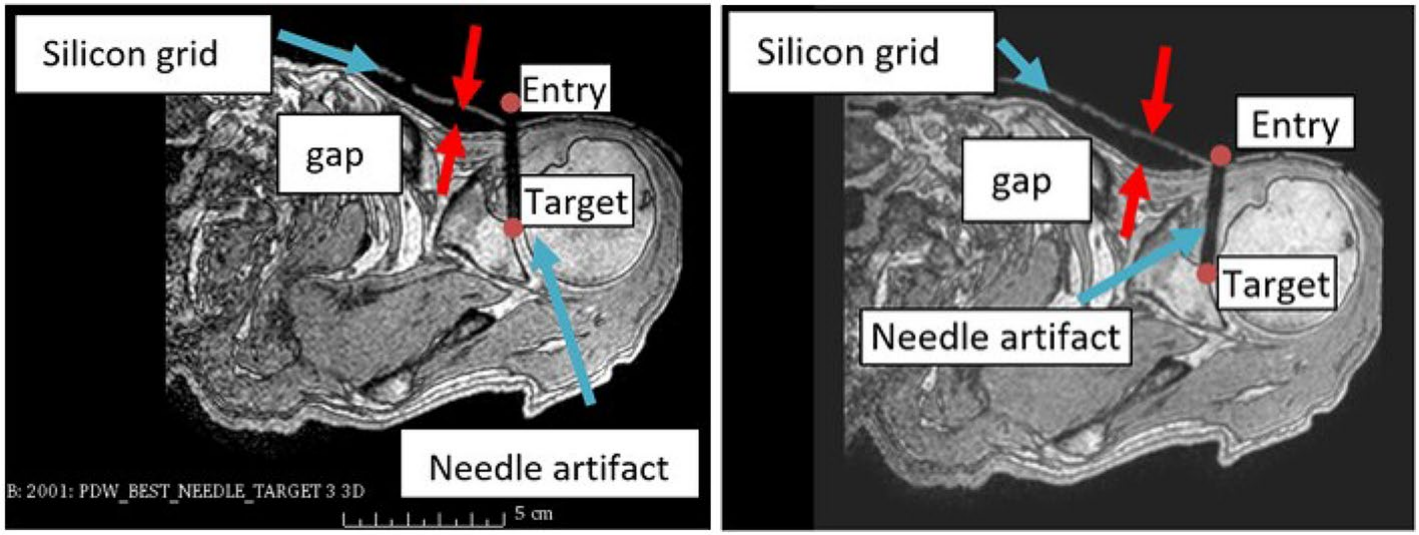
Confirmation image for targeting of joint shoulder in cadaver. T1-weighted MR imaging (T1, SP, Resolution 1 mm, FOV 200 × 200, TE1.79, TR4.43).

Figure 5 shows the MR image of the cadaver study setup. We conducted the targeting study on the left shoulder of the cadaver by selecting five different entry points and five different target points. After each planning and needle insertion, we acquired confirmation images (Fig. 5) to check the accuracy of the targeting. Targeting accuracy was calculated as the average normal distance between the planned target point and the needle.

To estimate the needle position on imaging, 10–20 points were manually selected along the center of the image artifact of the needle and a straight line was fitted to these points using MATLAB’s Singular Value Decomposition function. The Euclidean distance between this line, approximating the needle, and the target point selected by the radiologist was calculated for each targeting attempt. Table 1 shows the targeting accuracy for each replicate and the average targeting accuracy of 3.2 ± 1.0 mm.

**Table 1:** Shoulder joint targeting accuracy for five attempts.

Targets	Desired entry point (mm)	Desired target point (mm)	Normal target-to-needle distance (mm)
	(x, y, z)	(x, y, z)	
T1	(− 73.27, − 9.66, 38.39)	(− 75.82, − 46.77, 38.09)	4.8
T2	(− 76.34, − 9.19, 34.79)	(− 76.34, − 47.01, 34.79)	2.44
T3	(− 74.48, − 9.45, 18.59)	(− 77.39, − 45.62, 18.59)	3.70
T4	(− 68.57, − 8.20, 34.73)	(− 76.56, − 45.20, 34.79)	2.67
T5	(− 79.76, − 8.86, 32.09)	(− 76.98, − 48.53, 32.09)	2.50

## Discussion

The standard F2503-05 of the American Society of Testing and Materials (ASTM) categorizes devices in MRI environment into three different classes^4^; (1) MR-Safe: the device presents no additional risk to the patient or medical team when used in the MR environment, (2) MR-Conditional: the device when used in a specified MR environment (i.e. field strength, spatial gradient, time rate of change of the magnetic field, radio frequency fields, specific absorption rate) with specified conditions of use (i.e. specific configurations of the device) pose no known hazards to the patient or medical team, and (3) not MRI-safe. In this paper, we refer to the first two types of devices generally as “MRI-compatible” devices. In recent years, the MRI research community has focused on establishing new workflows, developing new devices, and robots to convert an MRI imaging room from a sole diagnosis room to an interventional suite, where diagnosis and intervention are done in the same room^5^. Real-time MR imaging technology has emerged in recent years as a valuable imaging tool that is made possible by modern MRI technology such as fast-switching gradients and parallel imaging^6^.

Using real-time MR imaging, a radiologist can select a target and introduce an MRI-compatible needle through the skin and with real-time visual feedback into the target anatomical structures, such as the joint space, to inject contrast agent or pain medicine^7,8^. The radiologist also can target different internal organs, such as prostate, breast, liver, and brain to perform biopsy and to detect cancerous lesions followed with a needle-based ablation procedures to burn or freeze malignant cells^9^.

MRI-compatible (MR-Safe or MR-Conditional) robots have been developed to reduce the burden on the clinician and improve the procedure accuracy. Different research groups have thus far focused on several technological aspects and anatomical structures^5^. Monfaredi et al. developed a patient-mounted robot for shoulder arthrography. This robot is a 4 degree of freedom (DOF) robot that is secured to the patient’s shoulder using a modified shoulder brace with an adaptor ring^10^. Another group in the Netherlands developed a 5-DOF pneumatically-actuated robotic system and tested it on 13 subjects. The mean biopsy error was larger when using the robotic technique compared to manual needle insertion (6.5 mm vs 4.4 mm)^11^. Mean procedure and manipulation durations were comparable. Larson et al developed the first surgical robotic device for real-time MRI-guided breast intervention in the United States^12^. Yang et al.^13^ reported the development of a master–slave breast biopsy robotic system using pneumatic cylinders to work in real-time MRI. Monfaredi et. Al. have published a comprehensive review paper that discusses recent advances in MRI-compatible robotics field^5^.

There are some needle guidance devices that use a grid mesh to provide a reference for entry point^14^. However, the simple repeating patterns used in these grids complicate the identification of the relationship between the grid and anatomy on imaging. This makes these solutions non-intuitive and cumbersome, potentially increasing the risk of missing the target and slowing trial and error approaches with multiple failed attempts. To eliminate exposure to ionizing radiation and to streamline the MRI arthrography procedure, we proposed and evaluated a novel MRI-safe Needle Guidance Toolkit.

The performance of the initial MRI arthrography kit prototype was originally evaluated using a gel phantom in a 1.5 T Philips Achieva MRI system to validate our proposed concept and to develop the clinical workflow. In this paper, we conducted a cadaver study to investigate the targeting accuracy of our toolkit in a more realistic scenario.

The average error for the cadaver targeting study is significantly higher than the phantom targeting study results of 2.2 ± 0.7 mm, previously conducted by our team^15^. The main reasons for increased error based on our observation are as follows: (1) the current silicone grid template conformed well to the flat gel surface in our earlier phantom study. However, in the cadaver study, the silicone template did not conform as well to the human shoulder curved anatomy, and there was a gap between the silicone template and the skin as shown in Fig. 5a,b, which resulted in deformation and displacement of the silicone during the needle insertion. (2) Due to the gap between skin and the silicone template, the template was stretched during the needle insertion. Therefore, the tip of the needle and the orientation of the needle changed after the needle was released from the needle guide partially contributing to the positioning error. As shown in Table 1 the targeting error was significantly reduced after the first attempt. Despite these issues, the accuracy of this method is better than the clinically required accuracy, i.e. 5 mm^16^.

Our proposed approach can be applied to both adult and pediatric populations for arthrography of different body joints. There are no differences in the workflow, patient anatomy, or trajectory planning between these age groups. In this study, an 84-year-old female specimen of small stature was used to represent an equivalent model for a teenage human and evaluated it for shoulder arthrography procedure. Similar studies could be conducted to evaluate the system for other joints.

This study did not require internal IRB approval. Based on our institute policy, the specimen should be provided from a certified institution that takes care of all the ethical and regulatory protocols. We received the specimen from Science Care Inc. which is recognized internationally and accredited by the American Association of Tissue Banks (AATB) after submitting our request and providing the justifications.

## Conclusion

We conducted a cadaver study to investigate the accuracy of our proposed MRI-based needle guidance toolkit. The device was tested by our interventional radiologist. The interventional radiologist selected several entry points on the skin and target points within the shoulder joint and used our proposed toolkit to plan and target the joint space.

The average targeting error was 3.2 $\pm$ 1.0 mm for this study. In the future, we will build the grid template with material and add finer patterns to the grid to increase the referencing accuracy. The silicone template will be fabricated so that one side of the silicone template is sticky. This will allow for the template to conform to the shape of the patient and avoid displacement of the grid template during needle insertion. For this study, we acquired two sets of MRI images for each targeting study, i.e. T1-weighted images and T2-weighted images. We will also develop a silicone grid that is visible in T2-weighted images to reduce the imaging time by eliminating T1-weighted imaging. The current hand-held device only covers a range of 6°. The primary objective of this study was to assess the visibility of the silicone template and to explore its feasibility for targeting purposes. The study's results demonstrated that the silicone template is indeed visible and provides sufficient detail for trajectory planning and targeting. The current handheld needle guide tool, in combination with the silicone grid template, proved effective in guiding the needle to the joint space. During this study, we noticed that the current handheld device with a range of 6° limits the angle of the trajectory thus the selectable entry point zone, and trajectories with less oblique angles were feasible. We are planning to improve the design to increase the range of angles to 35° to fix this limitation. In the longer term, we hope to conduct an IRB-approved human trial to study the usability of the device.

We conducted this study on the shoulder to investigate the performance of MR-arthrogram using our proposed MRI-safe system. This is in line with our goal to develop a clinical toolkit for MR-arthrogram of different anatomical joints of patients. This technology is not limited to pediatric applications and could be used for all age groups. At Children’s National Hospital, we are interested in conducting the first clinical studies on pediatric populations. However, we are planning to collaborate with researchers from an adult hospital to apply this technology to adult populations.

## Data Availability

The data that support the findings of this study are available on request from the corresponding author.

## Abbreviations

MRI: Magnetic resonance imaging
ASTM: American society of testing and materials
FV: Field of view
TE: Time of echo
TR: Time of relaxation
AATB: American association of tissue banks
KS: Interventional radiologist
